# Comprehensive analysis of diabetic nephropathy expression profile based on weighted gene co-expression network analysis algorithm

**DOI:** 10.1186/s12882-021-02447-2

**Published:** 2021-07-02

**Authors:** Alieh Gholaminejad, Mohammad Fathalipour, Amir Roointan

**Affiliations:** 1 Regenerative Medicine Research Center, Isfahan University of Medical Sciences, Isfahan, Iran; 2 Department of Pharmacology and Toxicology, Faculty of Pharmacy, Hormozgan University of Medical Sciences, Bandar Abbas, Iran

**Keywords:** Diabetic nephropathy, Weighted gene co-expression network, Transcriptome analysis, Key gene, Drug target

## Abstract

**Background:**

Diabetic nephropathy (DN) is the major complication of diabetes mellitus, and leading cause of end-stage renal disease. The underlying molecular mechanism of DN is not yet completely clear. The aim of this study was to analyze a DN microarray dataset using weighted gene co-expression network analysis (WGCNA) algorithm for better understanding of DN pathogenesis and exploring key genes in the disease progression.

**Methods:**

The identified differentially expressed genes (DEGs) in DN dataset GSE47183 were introduced to WGCNA algorithm to construct co-expression modules. STRING database was used for construction of Protein-protein interaction (PPI) networks of the genes in all modules and the hub genes were identified considering both the degree centrality in the PPI networks and the ranked lists of weighted networks. Gene ontology and Reactome pathway enrichment analyses were performed on each module to understand their involvement in the biological processes and pathways. Following validation of the hub genes in another DN dataset (GSE96804), their up-stream regulators, including microRNAs and transcription factors were predicted and a regulatory network comprising of all these molecules was constructed.

**Results:**

After normalization and analysis of the dataset, 2475 significant DEGs were identified and clustered into six different co-expression modules by WGCNA algorithm. Then, DEGs of each module were subjected to functional enrichment analyses and PPI network constructions. Metabolic processes, cell cycle control, and apoptosis were among the top enriched terms. In the next step, 23 hub genes were identified among the modules in genes and five of them, including FN1, SLC2A2, FABP1, EHHADH and PIPOX were validated in another DN dataset. In the regulatory network, FN1 was the most affected hub gene and mir-27a and REAL were recognized as two main upstream-regulators of the hub genes.

**Conclusions:**

The identified hub genes from the hearts of co-expression modules could widen our understanding of the DN development and might be of targets of future investigations, exploring their therapeutic potentials for treatment of this complicated disease.

## Background

Diabetic nephropathy (DN) is the main microvascular complication of diabetes mellitus and the major cause of end-stage renal disease [1]. Inflammatory processes, oxidative stress, overactive renin-angiotensin-aldosterone system (RAAS) and renal fibrosis are among the well-known pathogenesis features of DN [2]. Also, podocyte autophagy, mitochondria dysfunction, as well as some genetic and epigenetic modulations are among recently identified features of DN pathogenesis [3]. Despite such findings, current knowledge about the DN pathogenesis is not sufficient and treatment of this enigmatic disease is principally based on controlling the blood pressure, lowering the blood glucose, blocking the renin-angiotensin system and application of sodium/glucose cotransporter 2 inhibitors [4, 5]. Therefore, additional insights into the pathogenicity and genetic etiology of DN may offer new treatment options. Moreover, to have a precise and efficient treatment, there is a real need for discovery of novel therapeutic targets. In this context, exploring the biological variations at the genomic level would be a valuable strategy. During the pathogenesis of DN, numerous genes may subject to expressional alterations in a coordinated manner. Consequently, to describe and understand changes in gene expression profiles, genomic based approaches are needed [6]. Up to now, a majority of studies have been focused on the differential expression of genes associated with DN, but ignored their high degree of interconnectivity. In systems biology studies, weighted gene co-expression network analysis (WGCNA) has been effectively used to explore the intrinsic organization of transcripts [7]. The main aim of WGCNA is to simplify the interpretation of a huge number of genes with placing them in separate modules according to resemblances in their expression profiles. Sample clustering, construction of gene co-expression modules, and finally identifying hub genes based on their correlations with a trait are valuable features of this algorithm [8, 9].

The aim of this experiment was to perform WGCNA on DN samples not only to understand the disease related pathogenic pathways, but also to identify potential drug targets in this disorder. Accordingly, a DN related microarray dataset was downloaded from gene expression omnibus (GEO) database and its significant differentially expressed genes (DEGs) were identified. Subsequently, the DEGs were introduced to WGCNA in order to build the co-expression modules. Functional enrichment analyses showed the biological associations of co-expression modules with DN pathogenesis and after construction of protein-protein interaction (PPI) networks using genes in all the co-expression modules, hub genes were identified and validated in another DN dataset. To identify other regulatory molecules affecting the expression of hub genes, a multi-layer regulatory network comprising of hub gene’s interrelationships, the predicted miRNAs and transcription factors (TFs) was constructed and analyzed.

## Methods

### Data preprocessing and analysis

The microarray data from human glomeruli tissue samples of DN patients was downloaded as a part of GSE47183 dataset from GEO database (https://www.ncbi.nlm.nih.gov/gds). Analysis of the data was performed by networkanalyst online tool (http://www.networkanalyst.ca). Prior to data analysis, different filtering and normalization steps, including principal component analysis (PCA), variance stabilizing normalization and quantile normalization were performed to remove possible outliers and to make sure about the accuracy of the analysis. In addition, probes related to multiple genes were removed and for genes matching with multiple probes, the mean values of probes were considered as the gene’s expression values. The analysis procedure was done using Linear Model for Microarray Analysis (Limma) and significant DEGs were identified based on false discovery rate (FDR) cutoff <0.049. Volcano plot was built using R software (Version 1.2.5033).

### Construction of gene co-expression networks

Construction of co-expression network was performed using WGCNA algorithm. As a famous R software package, WGCNA is utilized for sample clustering, computation of topological features, co-expression network construction, selection of disease correlated genes and modules and differential analysis of networks [10]. Before WGCNA, outlier samples were recognized and removed using PCA method. Then, a matrix consisting of DEG’s related intensities for each sample was introduced to the WGCNA algorithm. After sample clustering, mean connectivity, as well as scale-free fit index for numbers 1–30 (as soft-threshold power (β)) were calculated separately, and the best value, which determines the adjacency matrix’s co-expression similarity, was recognized. Next, the calculated correlation matrix (based on Pearson’s correlation) was converted to adjacency matrix and the topological overlap matrix (TOM) was created, in which indirect relationships between genes are considered. Finally, using hierarchical clustering and TOM dissimilarity measures, all genes were classified into different modules (co-expression modules) according to their similarity in expression. In this step, after determining module eigengenes (based on Pearson’s correlation), the ones with highly correlated eigengenes (Pearson’s correlation higher than 0.85) were merged into one module. Identification of co-expression modules was performed using following parameters: “soft-threshold power = 12, minModuleSize = 30, mergeCutHeight = 0.15. In order to verify the modules division consistency, a heatmap was drawn for all genes to describe the adjacencies among them. In addition, the module interactions were shown by carrying out a cluster analysis and plotting the adjacency heatmap of eigengenes.

### Gene Ontology and pathway analysis

Gene ontology (GO) and pathway enrichment analyses were performed for the extracted genes from each co-expression modules. In this part, Cytoscape software (version 3.8.2) [11] and CluGO module (version 2.5.7) [12] were applied for biological process (BP), as well as Reactome pathway enrichment analyses. Revigo online tool (http://revigo.irb.hr/) was utilized to summarize and find the parent GO terms. The significant enrichment threshold was set as *p* < 0.05.

### Interactive network construction, hub gene analysis and identification

Search Tool for the Retrieval of Interacting Genes (STRING; version 11.0, combined score of >0.4)) [13] was utilized to determine interactions among the genes in each module. Cytoscape was applied for construction and visualization of PPI networks. Hub genes were selected based on both the degree centrality scores in the constructed PPI networks, as well as the weight scores in the weighted co-expression networks of the modules. In this part, at first top 5% of genes based on degree centrality scores in the PPI networks were identified using CytoHubba plugin [14] in Cytoscape. Likewise, the weighted co-expression networks were extracted from WGCNA algorithm and top 5% of genes based on their weight scores were achieved. The common genes in both lists were determined as hub genes.

### Hub gene validation and construction of a gene regulatory network

In order to verify the expression profiles of the identified hub genes and their levels of expression between normal and DN tissues, a different array dataset (GSE96804) was analyzed and checked. GSE96804 contained expression data from glomeruli samples of 41 DN patient and 20 healthy individuals [15]. In the next step, a multi-layer regulatory network was constructed to find other regulatory elements, including TFs and miRNAs affecting the expression of validated hub genes. In this part, the validated hub genes were uploaded into miRTarBase (Release 7) [16] and their associated miRNAs were extracted. The hub-gene associated TFs were also recognized in TRRUST (Version 2) database [17]. Finally, Cytoscape software was used for construction of a network comprising of hub genes, the predicted TFs, and miRNA molecules.

## Results

### Preprocessing, analysis, and identification of DEGs: 2475 DEGs were identified and subjected to further analysis

**Fig. 1:**
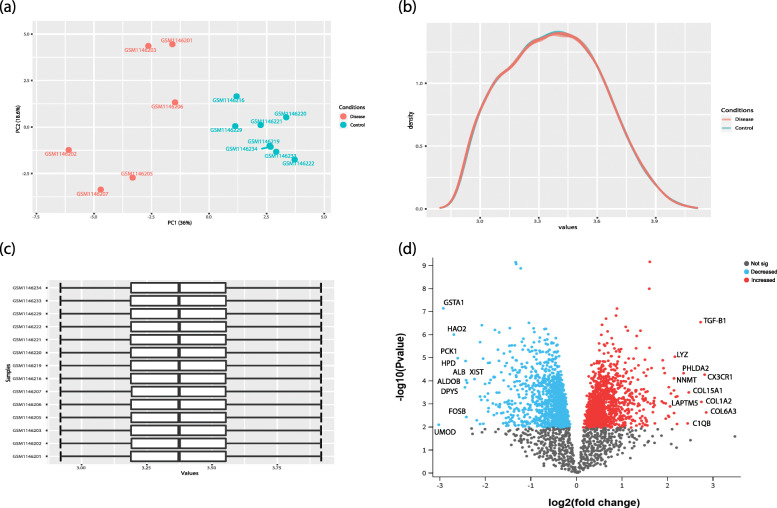
Filtering and normalization of the data before the analysis by Limma; All boxes showing the results of filtered and normalized dataset. (**a**) PCA plot representing the similarities and differences between the DN and control samples. (**b**) Plot of density against log2 of read counts showing relative distribution of different counts in each group. (**c**) Box plot showing the distribution of normalized samples. (**d**) Volcano plot of the analyzed dataset and top 10 up- and down-regulated genes

DN and control samples from the dataset GSE47183-GPL14663 included 7 DN and 14 control samples. PCA is known as a technique to explore similarities and differences of samples by reducing data dimensionality. PCA also could be a tool for showing dataset quality [18], and in a so-called good quality dataset, case and control samples are bunched together, separately. After performing PCA for the dataset, several samples, including one DN and six control samples were identified as outliers and removed from further analysis (Fig. 1a). Besides, prior to data analysis, two normalization procedures were performed to guarantee the similarity of the expression distributions of each sample across the entire dataset (Fig. 1b, c). Considering the FDR cutoff, 2475 significant DEGs, including 1183 down-regulated and 1323 up-regulated genes were selected for further analysis. Volcano plot representing the significant DEGs, as well as top 10 up- and down-regulated DEGs are shown in Fig. 1d.

### Construction of gene co-expression networks: genes were clustered into 6 co-expression modules

**Fig. 2:**
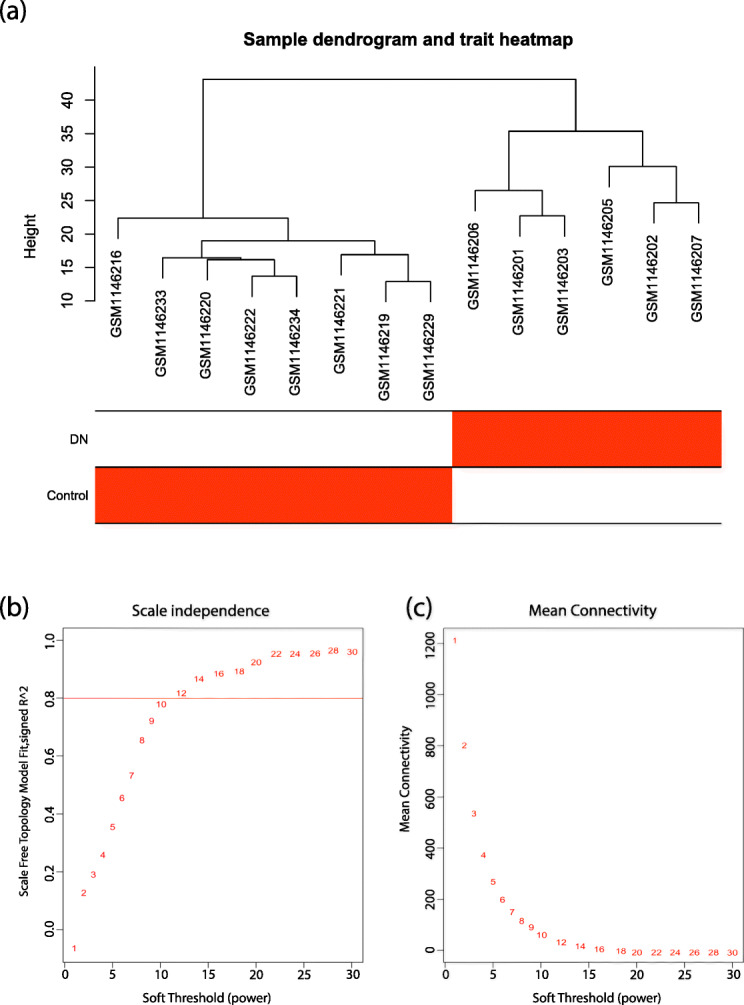
Sample clustering dendrogram, trait heatmap and soft-thresholding values. (**a**) Sample cluster dendrogram of 8 control and 6 DN samples. (**b**) Analysis of different soft-thresholding values from 1 to 30. (**c**) Evaluation of mean connectivity for each β value. β = 12 was selected for the sequential analyses for which both the mean connectivity and scale-free topology fitting index R2 may reach a plateau

**Fig. 3:**
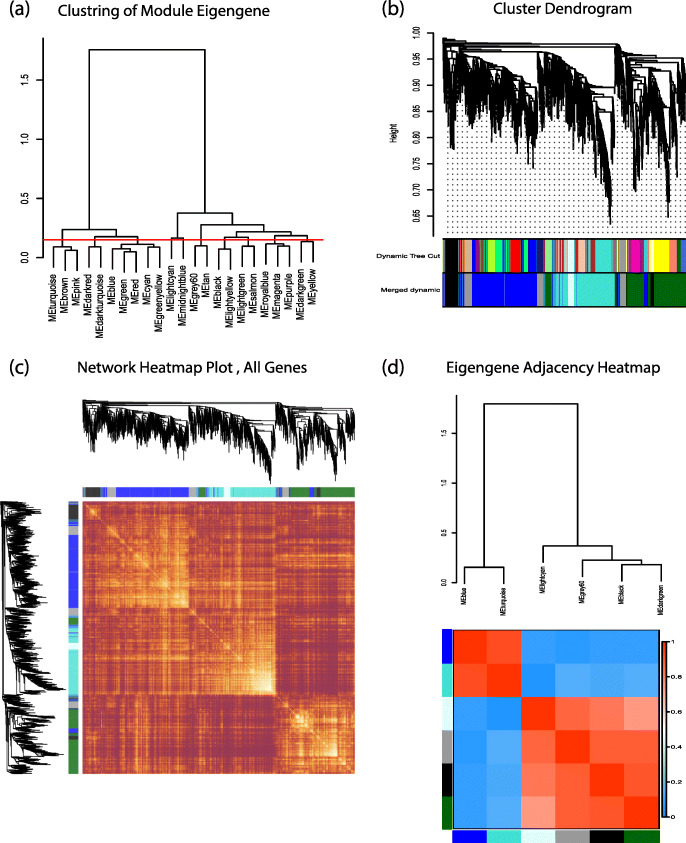
Construction and validation of co-expression modules using WGCNA algorithm. (**a**) Hierarchical cluster analysis of the genes in different modules. The horizontal red line represents the threshold (0.15) used for merging the modules. (**b**) Cluster dendrogram of all genes classified in different modules according to the dissimilarity measure. The colored bars below the dendrogram represent the original division of modules based on hierarchical clustering (upper bar), and the merged modules based on eigengenes Pearson’s correlation (lower bar). (**c**) Adjacency heatmap of all genes, indicating the accuracy of the module division. Each row and column of the heatmap belong to a single gene. Red color indicates low adjacencies and progressive yellow color indicate higher adjacencies among genes in the modules. (**d**) Clustering dendrogram and adjacency heatmap of eigengenes. Red indicated positive correlation and blue indicated negative correlation between co-expression modules

Sample clustering showed no outlier among samples (Fig. 2a) and soft-threshold power of 12 was selected based on the scale-free fit index and mean connectivity values (Fig. 2b, c). WGCNA algorithm clustered genes into six co-expression modules, including black, blue, turquoise, grey, dark-green, and light cyan modules (Fig. 3a, b). The number of genes in each module is shown in Table 1. The plotted heatmap revealed the module division accuracy and the topological overlap adjacency among genes in the modules. In the heatmap, most of the genes in the same module have a higher correlation (Fig. 3c). According to the eigengene’s clustering dendrogram and adjacency heatmap, six co-expression modules were divided into two clusters (Fig. 3d).

**Table 1:** The number of genes and the identified hub genes in each co-expression module

Module	Genes	Hub genes/validated hub genes
Blue	781	PTEN, ESR1, PSMA5, RPS9, PSMC4, NEDD8, RPL3
Black	165	PECAM1
Dark-green	590	FN1^★^, CDC20, AURKB, MAD2L1, RAD51, UBA52, AURKA,
Grey	196	GAPDH
Light cyan	60	–
Turquoise	683	EHHADH^★^, PIPOX^★^, SLC2A2^★^, FABP1^★^, HADH, ACADM, ACAA1

★Validated hub genes in another DN dataset (GSE96804)

### Functional analysis of genes in co-expressed modules: metabolic processes, cell cycle processes and apoptosis were top enriched terms

GO terms of biological process and Reactome pathways were recognized for the genes in each co-expression module. The light cyan module was not considered for further analysis due to the small number of genes and no significant enrichment results. Top five enriched GO terms and Reactome pathways (*p*-value <0.05) are shown in Table 2. Genes in the black module were mostly enriched in regulation of ATP metabolic process, glycolytic process, purine nucleotide metabolic process, skeletal muscle tissue development, and blood vessel endothelial cell migration. In terms of pathway enrichment, genes in this module were enriched in collagen biosynthesis, ERK/MAPK targets and Interleukin-17 signaling. The top BP terms of the blue module with 781 genes were the regulation of the multicellular organismal process, regulation of growth, and transcription initiation. In addition, in terms of pathway enrichment analysis, genes in the blue module were mainly enriched in programmed cell death and apoptosis, transcriptional regulation by RUNX2, UCH proteinases, and regulation of ornithine decarboxylase (ODC).

**Table 2:** The results of functional analyses for genes in each module. Top 5 GO terms and Reactome pathways were listed

Module	Enrichment	Term	Count	% Associated Genes	*p*-value
Black	GO	GO:1903579- negative regulation of ATP metabolic process	4	15.38461	6.87E − 05
		GO:0045820- negative regulation of glycolytic process	3	21.42857	2.14E − 04
		GO:0043534- blood vessel endothelial cell migration	6	4.918033	6.64E − 04
		GO:1900543- negative regulation of purine nucleotide metabolic process	3	14.28571	7.47E − 04
		GO:0048643- positive regulation of skeletal muscle tissue development	3	14.28571	7.47E − 04
	Reactome	R-HSA:1650814- Collagen biosynthesis and modifying enzymes	5	7.462687	6.61E − 04
		R-HSA:198753- ERK/MAPK targets	3	13.63636	0.001484
		R-HSA:448424- Interleukin-17 signaling	3	4.166667	0.039484
Blue	GO	GO:0051239- regulation of multicellular organismal process	194	5.687482	2.76E − 10
		GO:2000026- regulation of multicellular organismal development	139	6.264083	5.13E − 10
		GO:0006367- transcription initiation from RNA polymerase II promoter	28	13.46154	4.33E − 09
		GO:0040008- regulation of growth	58	8.011049	5.86E − 08
		GO:0006352- DNA-templated transcription, initiation	30	10.9489	1.46E − 07
	Reactome	R-HSA:109581- Apoptosis	24	13.33333	5.58E − 07
		R-HSA:5357801- Programmed Cell Death	25	12.7551	7.57E − 07
		R-HSA:8878166- Transcriptional regulation by RUNX2	18	14.87603	3.04E − 06
		R-HSA:5689603- UCH proteinases	16	15.68627	5.34E − 06
		R-HSA:350562- Regulation of ornithine decarboxylase (ODC)	11	21.56863	7.06E − 06
Dark-green	GO	GO:0022402- cell cycle process	96	6.521739	6.93E − 15
		GO:1903047- mitotic cell cycle process	71	7.768053	7.68E − 15
		GO:0042981- regulation of apoptotic process	96	6.185567	1.62E − 13
		GO:0010564- regulation of cell cycle process	63	7.758621	3.17E − 13
		GO:0051246- regulation of protein metabolic process	130	4.708439	1.56E − 09
	Reactome	R-HSA:156842- Eukaryotic Translation Elongation	17	18.27957	1.14E − 08
		R-HSA:72764- Eukaryotic Translation Termination	17	18.27957	1.14E − 08
		R-HSA:975956- Nonsense Mediated Decay (NMD) independent of the Exon Junction Complex (EJC)	17	17.89474	1.59E − 08
		R-HSA:69278- Cell Cycle, Mitotic	46	8.199643	2.13E − 08
		R-HSA:156902- Peptide chain elongation	16	17.97753	3.95E − 08
Grey	GO	GO:0051014- actin filament severing	4	21.05263	3.80E − 05
		GO:0036498- IRE1-mediated unfolded protein response	6	8.450705	9.25E − 05
		GO:1902749- regulation of cell cycle G2/M phase transition	10	4.329004	1.50E − 04
		GO:0002479- antigen processing and presentation of exogenous peptide antigen via MHC class I, TAP-dependent	6	7.5	1.80E − 04
		GO:0034378- chylomicron assembly	3	23.07692	2.88E − 04
	Reactome	R-HSA:69601- Ubiquitin Mediated Degradation of Phosphorylated Cdc25A	6	11.53846	6.09E − 05
		R-HSA:69610- p53-Independent DNA Damage Response	6	11.53846	6.09E − 05
		R-HSA:5358346- Hedgehog ligand biogenesis	6	9.230769	2.14E − 04
		R-HSA:8963888- Chylomicron assembly	3	30	2.57E − 04
		R-HSA:69481- G2/M Checkpoints	9	5.357143	3.95E − 04
Turquoise	GO	GO:0006082- organic acid metabolic process	138	11.89655	2.38E − 54
		GO:0044282- small molecule catabolic process	84	17.46362	4.44E − 45
		GO:0032787- monocarboxylic acid metabolic process	82	11.61473	1.27E − 30
		GO:0006629- lipid metabolic process	96	6.241873	9.88E − 16
		GO:0019395- fatty acid oxidation	24	20.33898	4.86E − 15
	Reactome	R-HSA:1430728- Metabolism	166	7.844991	4.14E − 29
		R-HSA:71291- Metabolism of amino acids and derivatives	49	13.1016	2.81E − 16
		R-HSA:8978868- Fatty acid metabolism	30	16.94915	2.79E − 13
		R-HSA:211859- Biological oxidations	31	13.96396	2.16E − 11
		R-HSA:390918- Peroxisomal lipid metabolism	12	41.37931	6.80E − 11

Genes in the dark-green module were mostly enriched in the cell cycle process, protein metabolic process, as well as pathways like translation, cell cycle and nonsense-mediated decay (NMD) independent of the exon junction complex (EJC). GO terms of genes in the grey module included actin filament severing, IRE1-mediated unfolded protein response, regulation of cell cycle G2/M phase transition, chylomicron assembly, antigen processing and presentation of exogenous peptide antigen. In terms of pathway enrichment, the grey module genes were enriched in G2/M checkpoints, ubiquitin-mediated degradation of phosphorylated Cdc25A, p53-independent DNA damage response, hedgehog ligand biogenesis and chylomicron assembly.

In the turquoise module, genes were mostly enriched in metabolic processes like organic acid metabolic process, small molecule catabolic process, monocarboxylic acid metabolic process, lipid metabolic process, and fatty acid oxidation. In terms of pathway enrichment, genes of this module were involved in the metabolism of amino acids and derivatives, fatty acid metabolism, biological oxidations and peroxisomal lipid metabolism.

### Hub gene identification, and validation: 23 hub genes were identified and 5 of them were validated in another DN dataset

**Fig. 4:**
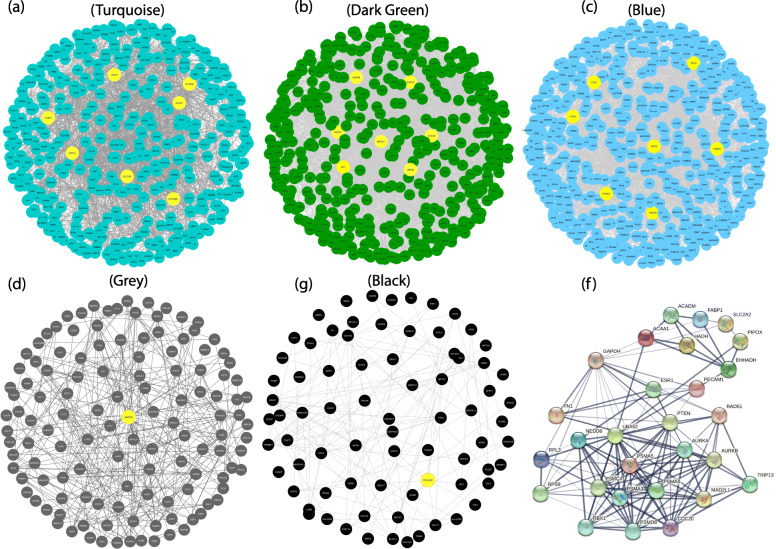
The constructed PPI network by genes of the co-expressed modules (**a**–**e**). The nodes in yellow represents identified hub genes. The hub genes are the ones that listed as top genes in the co-expression networks and have the highest degree centrality in the PPI networks. (**f**). PPI network of all the hub genes based on STRING database. All the hub genes from different co-expression modules are closely connected together in the constructed PPI network

**Fig. 5:**
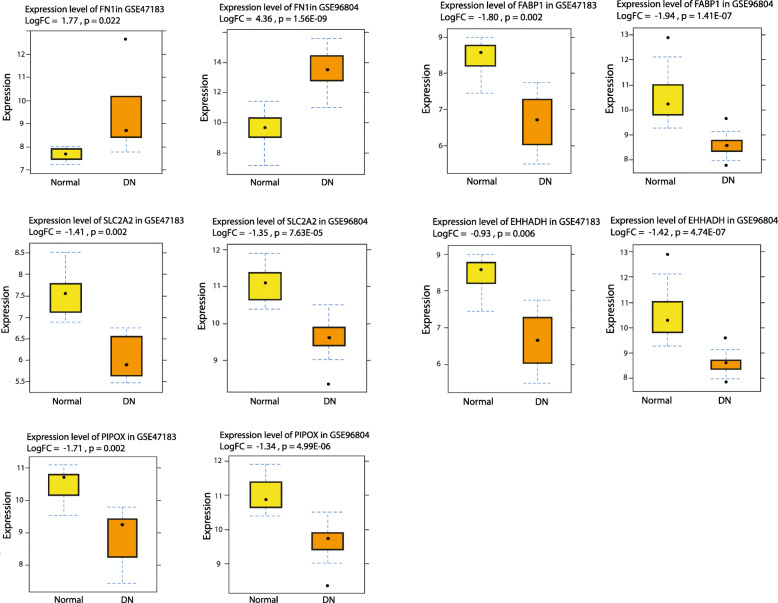
Expression levels of FN1, SLC2A2, PIPOX, FABP1, and EHHADH in normal and DN samples in the main (GSE47183) and validation (GSE96804) datasets

Genes in all co-expression modules were extracted and their PPI networks were constructed using the STRING database (Fig. 4a–e). Instead of genes in the light cyan module, the extracted genes from the other five co-expression modules showed a close interaction in the constructed PPI networks. The hub genes were identified from the top 5% of the PPI-related hub genes (based on degree centrality) and the top 5% of the weighted networks for each module. The identified hub genes in 5 modules were including: ACAA1, ACADM, AURKA, AURKB, CDC20, EHHADH, ESR1, FABP1, FN1, GAPDH, HADH, MAD2L1, NEDD8, PECAM1, PIPOX, PSMA5, PSMC4, PTEN, RAD51, RPL3, RPS9, SLC2A2, and UBA52. Notably, all the identified hub genes were connected together in an interactive network and a close correlation was observed between RPL3, PSMA5, MAD2L1, PSMC4, PTEN, NEDD8, AURKA, UBA52, AURKB, CDC20, GAPDH, and RPS9 (Fig. 4f). In order to verify the differentially expressed profile of the hub genes in another DN-related dataset, GSE96804 was downloaded and analyzed. Five of the 23 identified hub genes, including: FN1, SLC2A2, PIPOX, FABP1, and EHHADH showed similar up/down-regulation patterns with close log2 fold change in the validation dataset. The expression profiles of these five hub genes are shown in Fig. 5. Instead of FN1, all other hub genes were found to be downregulated in DN samples.

### Construction of multi-layer regulatory network: hsa-miR-27a-3p and RELA were predicted as top upstream regulators of the validated hub genes

**Fig. 6:**
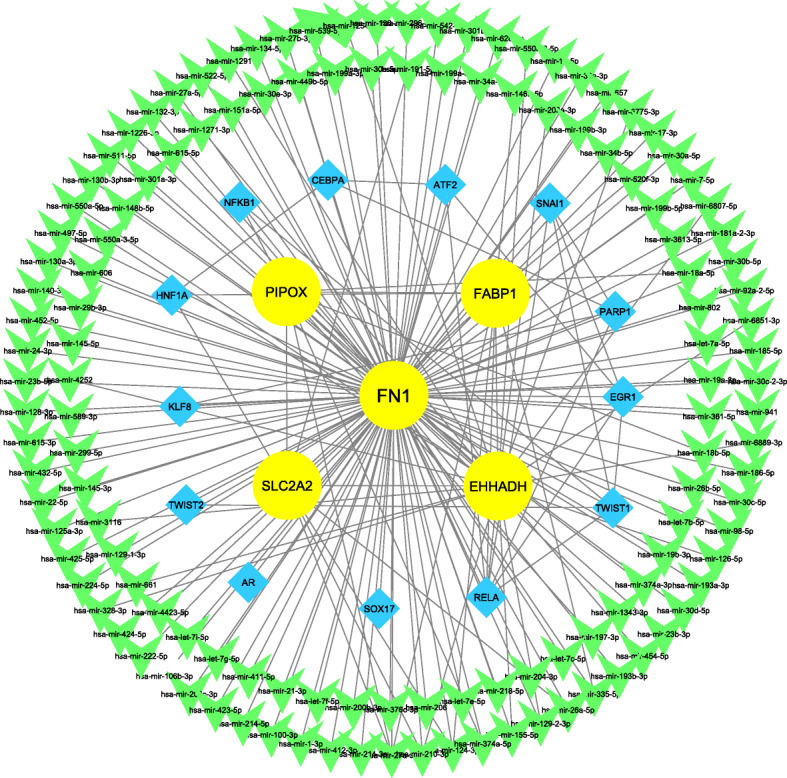
The multilayer gene regulatory network comprising of 5 validated hub genes, and their related miRNAs and transcription factors. Among the hub genes, FN1 was the most affected gene by both regulatory layers. MiR-27a and RELA were two regulatory elements affecting most of the hub genes

In the next step, in order to identify other regulatory elements affecting the expression of validated hub genes, a multi-layer regulatory network comprising of hub gene’s interrelationships, predicted miRNAs, and TFs was constructed and analyzed (Fig. 6). The constructed regulatory network comprised of 148 nodes, including five hub genes, 130 miRNA, and 13 TFs. According to the degree of connectivity, hsa-miR-27a-3p and RELA were recognized as top potential up-stream regulators, affecting the expression of the validated hub genes. In addition, FN1 was recognized as the most affected gene by both the TF and miRNA molecules.

## Discussion

The aim of this study was to identify underlying molecular pathways and key genes in the pathogenesis of DN using the WGCNA algorithm. Compared to conventional microarray-based profile analysis, WGCNA has some distinct advantages, like considering gene clusters (modules), rather than analyzing whole genes and their interactions. As a general hypothesis, genes within a co-expressed module are more likely under the control of similar regulatory pathways. These cluster of genes could also most likely be functionally related. Therefore, identification of the genes with co-expressed profiles is beneficial in determining the most disease-associated genes and their functions [7].

According to the results of enrichment analysis, the turquoise module was more likely associated with the DN pathogenesis. Some of the DN explicit physiological pathways, including fatty acid and peroxisomal lipid metabolism, amino acid metabolism, and biological oxidations were among the top enriched ones for the genes in this module. Based on recent findings, alteration in metabolite levels in some pathways, including the TCA cycle, lipids metabolism, amino acids metabolism, and the urea cycle, is strongly associated with DN progression [19–22]. Aberrant levels of metabolites are also linked with oxidative stress and changes in renal hemodynamics. Moreover, oxidative stress has been shown to play a significant role in podocyte damage, proteinuria, and tubulointerstitial fibrosis [23, 24].

Other enriched terms and pathways for genes in the black, blue, dark-green, and grey modules also were connected with the pathogenesis of DN. For instance, collagen biosynthesis, ERK/MAPK and interleukin-17 signaling pathways that were top enriched pathways for black module genes, have shown to play important roles in renal pathogenesis and fibrosis [25–27]. Likewise, apoptosis as the top enriched term for the blue module genes, has been previously found in tubular, epithelial, endothelial, and interstitial cells of DN patients [23]. Cell cycle regulation was another main enriched term for the genes in dark-green and gray modules. Although, the cell cycle machinery and elements are the same in all cells, in some tissues like kidney, this machinery might be under the control of some distinct growth factors able to cause different growth responses based on the cell types. For instance, transforming growth factor-β (TGF-β), which is a well-known molecule in DN progression, stimulates the propagation of tubulointerstitial fibroblasts, but also invoke hypertrophy in some other cells like mesangial and tubular cells [28, 29]. As another example, angiotensin II stimulates the propagation of fibroblasts, mesangium cells, and distal tubular cells, but also intermediating hypertrophy in proximal tubules [30, 31]. Such behaviors might be due to the differential expression of growth factor receptors during the DN development [28]. Generally, cell turnover in the normal kidney is low, and most of the cells are in the G0 phase of the cell cycle. Following injury, both the cell division and hypertrophy as compensatory mechanisms will be initiated to stop organ dysfunction [32]. Therefore, during the DN development, it is thought that the regulatory elements participating in the cell cycle control are in their high activity levels. Discovery of the key mediators and understanding their detailed roles in the cell cycle pathway could be valuable in the development of novel therapeutic strategies aimed for clogging the DN progression.

Among the validated hub genes, fibronectin 1 (FN1), was the only one that showed an up-regulated pattern in DN samples. This non-collagenous glycoprotein is one of the principal components of the extra-cellular matrix (ECM), playing an important role in both cell-cell and cell-matrix interactions. So far, a great number of experiments have pointed to the key mediatory role of FN1 in glomerular sclerosis and fibrosis in different chronic kidney diseases (CKDs) [33–36]. The process of FN matrix assembly is a step-by-step process started by α5β1 integrin receptors. Generally, these receptors result in FN-FN interactions and the formation of the nascent fibrils [37]. Then, the fibrils grow into a mature and insoluble matrix acting as a basement for the deposition of other ECM components like collagens. Consequently, FN1 dysregulation could have disturbing effects on the organization, quantity, and structure of ECM fibrils launching a fibrotic response [38]. Moreover, due to the slow turnover of ECM, the situation could cause harmful effects on the glomerulus filtration [39]. Therefore, controlling FN assembly might be an accurate strategy in targeting ECM accumulation during kidney fibrosis. FN could also be considered as a biomarker in different CKDs. During fibrosis, both the circulatory FN and local FN have shown to be up-regulated in Bowman’s capsule, tubule-interstitium and glomerular mesangium [40]. Thus, since the degree of fibrosis is a great indicator of renal function in kidney diseases, FN could be considered as an explicit biomarker of fibrosis and a potential progression indicator in CKDs like DN.

Four other hub genes, including SLC2A2, PIPOX, FABP1 and EHHADH were among the co-expressed genes in the turquoise module, also down-regulated genes in DN samples. Notably, these DEGs are involved in some DN explicit physiological features, such as lipids and glucose metabolism (EHHADH, FABP1, SLC2A2) also oxidation-reduction process (PIPOX) [41, 42].

Flavoenzyme pipecolate oxidase or PIPOX is responsible for the oxidation of L-pipecolic acid to Δ1-piperideine-6-carboxylate (P6C) [43]. Pipecolate is a nonproteinogenic amino acid of lysine metabolism and its metabolism consequently results in a protection against H2O2 stress [44] . Since PIPOX is required in this process, down-regulation of this enzyme in DN could reduce the protective effects of L-pipecolic acid against oxidative stress pathways and accelerate the disease progression [45].

Glucose transporter 2 (GLUT2) (encoded by *SLC2A2* gene) is a high-capacity facilitative glucose transporter expressed in different organs, including liver, kidney, intestine, and pancreatic β-cells [1]. In the kidney, GLUT2 maintains glucose homeostasis by regulating the transepithelial uptake of glucose in the epithelial cells of the basolateral membrane, as well as glucose reabsorption in the kidney proximal tubule [46]. As a glucose transporter, GLUT2 might play a role in insulin signaling and glucose uptake in podocytes. But, in diabetic condition, podocytes use different types of transporters for glucose uptake. Based on an investigation, high glucose concentrations and mechanical stress could reduce the expression of GLUT2 and GLUT4, while enhancing glucose uptake in rat podocytes [47]. The present study also revealed the downregulation of GLUT2 in DN samples and introduced this transporter as a hub gene. However, upregulation of this transporter and the altered glucose uptake in hyperglycemia condition and DN models have been shown in different studies [48]. It seems that more investigations are required to clarify the expressional pattern, as well as the concealed roles of this transporter in DN pathogenicity.

Fatty acid-binding protein 1, FABP1, was another identified hub gene with a reduced expression in DN samples. FABP1, which is mainly expressed in the liver, is responsible for the metabolism of long-chain fatty acids and other hydrophobic molecules [49]. FABP1 is also expressed in kidneys, mostly in the cytoplasm of kidney proximal tubule cells [50]. According to previous investigations, some conditions like hyperglycemia, hypertension, proteinuria, and toxin-induced damage to kidney proximal tubule cells could increase the urinary excretion of FABP1 [51, 52] and consequently, reduce renal levels of this protein. Moreover, FABP1 has been shown to play a central role in kidney damage and repair processes; Therefore, its urinary concentration might be a potential indicator for prediction of DN occurrence and severity [53]. Based on other findings, overexpression of FABP1 in proximal tubule cells can decrease angiotensin II-induced oxidative stress and tubulointerstitial damage [54].

Another identified hub gene was enoyl-CoA hydratase and 3-hydroxyacyl CoA dehydrogenase (EHHADH), which is a part of the classical peroxisomal fatty acid β-oxidation pathway. Generally, decreased levels of this protein might lead to the accumulation of lipids in tubular epithelial cells, which is strongly linked with decreased renal function [41, 55]. In a normal state, tubular epithelial cells depend on fatty acids as the main source of energy, whereas faulty utilization of fatty acids leads to energy depletion. Since, the baseline energy consumption of tubular epithelial cells is high, the resulted energy depletion would finally cause excessive oxidative stress, cell damage, and cell death [56, 57].

TFs and miRNAs as two main types of regulatory elements are controlling the expression of genes. By construction of a regulatory network, miR-27a-3p and RELA were recognized as potential top molecules affecting the expression of the five identified hub genes. The members of miR-27 family usually contributes to the regulation of cell cycle progression, cell proliferation and cell hypertrophy [58, 59]. Upregulation of miR-27a was shown in cultured glomerular mesangial cells and in kidney glomeruli of streptozotocin-induced diabetic rats. Moreover, inhibition of this miRNA was shown to reduce mesangial cell proliferation and ECM accumulation, along with triggering some necessary pathways for recovery from kidney injury [60]. Likewise, inhibition of this miRNA in the kidney of db/db mice was shown to reverse mitochondrial dysfunction by affecting mitochondrial membrane potential and production level of reactive oxygen species [61]. Among the 5 validated hub-genes, PIPOX, FABP1, and SLC2A2 were identified as the downregulated DEGs in DN cases and targets of mir-27a. FN1 is also recognized as another target of miR-27a, but with an upregulated profile in DN cases. It can be assumed that the expression of FN1 is under the control of other regulatory elements not just the miR27a.

RELA, also known as p65, was identified as the top TF having connections with other TFs and affecting the expression of FN1, FABP1 and EHHADH in the constructed regulatory network. This TF is a REL-associated protein involved in the formation of nuclear factor NF-kappa-B (NF-κB) heterodimer, which is an essential complex in various cellular processes, such as cellular metabolism and chemotaxis [62]. Activation of NF-κB and subsequent production of certain pro-inflammatory chemokines in tubular epithelial cells are indicators of progressive DN [63]. This might point to the potential role of RELA in DN pathogenesis.

## Conclusions

The main strength of this study was to consider both the degree centrality values in PPI networks, as well as the weighted networks for the identification of key players in the DN pathogenesis. One limitation of this study was the lack of an experimental section checking the expression of the identified hub genes in DN samples. This issue was partially covered by performing validation in another DN dataset. Due to the lack of diabetic (without nephropathy), as well as non-diabetic nephropathy samples as control samples, we only consider the comparison of DN versus healthy controls. This could be another limitation of this work. All in all, these data may lead to future experimental studies examining the role of the spotted genes in the pathophysiology of DN. Additionally, the identified genes and their involved pathways could widen our understanding of the DN development and might be targets of future investigations, exploring their therapeutic potentials for treatment of this complicated disease.

## Data Availability

The analyzed dataset by the present study is available in the GEO repository, [https://www.ncbi.nlm.nih.gov/geo/query/acc.cgi?acc=GSE47183].
